# Preliminary study of ventilation with 4 ml/kg tidal volume in acute respiratory distress syndrome: feasibility and effects on cyclic recruitment - derecruitment and hyperinflation

**DOI:** 10.1186/cc12487

**Published:** 2013-01-28

**Authors:** Jaime Retamal, Javiera Libuy, Magdalena Jiménez, Matías Delgado, Cecilia Besa, Guillermo Bugedo, Alejandro Bruhn

**Affiliations:** 1 Departamento de Medicina Intensiva, Facultad de Medicina, Pontificia Universidad Católica de Chile, Marcoleta 367, 3º piso, Santiago, PO Box 114-D, Chile; 2 Departamento de Radiología, Facultad de Medicina, Pontificia Universidad Católica de Chile, Marcoleta 367, 2º piso, Santiago, PO Box 114-D, Chile

## Abstract

**Introduction:**

Cyclic recruitment-derecruitment and overdistension contribute to ventilator-induced lung injury. Tidal volume (Vt) may influence both, cyclic recruitment-derecruitment and overdistension. The goal of this study was to determine if decreasing Vt from 6 to 4 ml/kg reduces cyclic recruitment-derecruitment and hyperinflation, and if it is possible to avoid severe hypercapnia.

**Methods:**

Patients with pulmonary acute respiratory distress syndrome (ARDS) were included in a crossover study with two Vt levels: 6 and 4 ml/kg. The protocol had two parts: one bedside and other at the CT room. To avoid severe hypercapnia in the 4 ml/kg arm, we replaced the heat and moisture exchange filter by a heated humidifier, and respiratory rate was increased to keep minute ventilation constant. Data on lung mechanics and gas exchange were taken at baseline and after 30 minutes at each Vt (bedside). Thereafter, a dynamic CT (4 images/sec for 8 sec) was taken at each Vt at a fixed transverse region between the middle and lower third of the lungs. Afterward, CT images were analyzed and cyclic recruitment-derecruitment was determined as non-aerated tissue variation between inspiration and expiration, and hyperinflation as maximal hyperinflated tissue at end-inspiration, expressed as % of lung tissue weight.

**Results:**

We analyzed 10 patients. Decreasing Vt from 6 to 4 ml/kg consistently decreased cyclic recruitment-derecruitment from 3.6 (2.5 to 5.7) % to 2.9 (0.9 to 4.7) % (*P *<0.01) and end-inspiratory hyperinflation from 0.7 (0.3 to 2.2) to 0.6 (0.2 to 1.7) % (*P *= 0.01). No patient developed severe respiratory acidosis or severe hypercapnia when decreasing Vt to 4 ml/kg (pH 7.29 (7.21 to 7.46); PaCO2 48 (26 to 51) mmHg).

**Conclusions:**

Decreasing Vt from 6 to 4 ml/kg reduces cyclic recruitment-derecruitment and hyperinflation. Severe respiratory acidosis may be effectively prevented by decreasing instrumental dead space and by increasing respiratory rate.

## Introduction

The main mechanisms of ventilator-induced lung injury (VILI) are overdistension and cyclic recruitment-derecruitment (R/D). While low tidal volume (Vt) has been applied successfully with the rationale of preventing overdistension [1], high positive end-expiratory pressure (PEEP) strategies, which should theoretically help to prevent cyclic R/D, have not been able to show a clear benefit on survival in acute respiratory distress (ARDS) patients [2-4]. One of the main drawbacks of increasing PEEP to prevent cyclic R/D is that a parallel increase in stress and strain occurs at end-inspiration.

We recently showed that Vt is also an important determinant of cyclic R/D, and that high Vt may induce injury not only by inducing overdistension, but also cyclic R/D. However, we observed that at a Vt of 6 ml/kg there was still evidence of cyclic R/D [5]. Other studies have also shown that a Vt of 6 ml/kg may still be associated to hyperinflation, which has been shown to be a relevant marker of VILI [6,7]. Therefore, it has been proposed that in order to achieve higher levels of protection, it may be necessary to move toward ultra-protective strategies [6,8,9]. High-frequency oscillatory ventilation or extracorporeal lung support techniques have been suggested as potential alternatives in this direction. However, these interventions require rather complex technologies, not widely available, and which have still not proven to be superior to conventional protective mechanical ventilation.

An alternative strategy may be to optimize conventional protective mechanical ventilation by further decreasing Vt below 6 ml/kg. Based on our previous findings, we hypothesize that this intervention may further decrease cyclic R/D, but in contrast to increasing PEEP, a lower Vt may also decrease the risk of overdistension. However, an important concern with using very low Vt is the risk of severe hypercapnia and respiratory acidosis.

The goal of this preliminary physiologic study was to determine if decreasing Vt from 6 to 4 ml/kg, with conventional mechanical ventilation, is effective to reduce cyclic R/D and hyperinflation, and if it is possible to prevent severe hypercapnia and respiratory acidosis at such very low Vt, without using extracorporeal lung support techniques.

## Materials and methods

We designed a crossover study at two Vt levels: 6 and 4 ml/kg predicted body weight (PBW). The protocol had two parts: one at the bedside and other at the computed tomography (CT) room. The Research Ethics Committee of the School of Medicine of the Universidad Católica de Chile granted approval for this study, and informed consent was obtained from the patients' next of kin. We included patients with acute onset of hypoxemia (PaO2/FiO2 <300), bilateral opacities in chest X-ray, without evidence of left ventricle overload, connected to mechanical ventilation for less than 48 hours, and who required a lung CT scan according to the treating physician.

### Ventilatory protocol

At baseline all patients were ventilated in assist - control mode, with a heat and moisture exchanging filter (HMEF, compressible volume = 57 ml), under deep sedation and neuromuscular paralysis. The first part of the protocol took part at the ICU (ICU protocol). Starting from usual ventilatory care, patients were ventilated in a random order with two different settings for 30 minutes each: (a) Vt 6 with Vt 6 ml/kg PBW, and (b) Vt 4 with Vt 4 ml/kg PBW. Respiratory rates at each setting were calculated to keep minute ventilation constant and identical to baseline, despite the change in Vt values. Total PEEP was measured in all patients at 4 and 6 ml/kg after a 2-second end-expiratory pause. For both settings I:E time ratio was kept at 1:2, inspiratory pause at 10%. PEEP and FiO_2 _were kept at baseline levels and were not modified during the protocol. Following the general protocol of the ICU, PEEP was set according to best compliance after an initial recruitment maneuver of 40 cmH_2_0 for 40 seconds, followed by a decremental PEEP trial [10]. Although an HMEF was used routinely in our ICU unless severe hypercapnia was present, for the 4 ml/kg arm (but not for the 6ml/kg arm) HMEF was replaced by a heated humidifier to compensate for the increase in dead space [11]. Based on previous trials, which applied permissive hypercapnia, severe respiratory acidosis was defined as pH <7.2 and severe hypercapnia as PaCO_2 _>80 mmHg [12,13]. For safety reasons we predefined that the protocol could be stopped at any moment if marked alterations in systemic hemodynamics were observed.

Thereafter, patients were transferred to the CT room. After performing a conventional whole lung CT scan (LightSpeed VCT, GE Healthcare, Amersham, UK), a 1.25 mm-thick CT slice, between the middle and lower third of the thorax, was selected for the dynamic protocol (CT protocol).

### Dynamic CT protocol

As described elsewhere [5], CT was set in cine mode at fixed position with a matrix of 512 × 512, at a velocity of 0.4 sec/rotation, for 8 seconds, with image reconstruction every 250 msec, which resulted in 32 consecutive images. A cine-CT was performed at each ventilatory setting, without interrupting ventilation, following the same crossover sequence as the ICU protocol, and after a short period of stabilization of 5 to 10 minutes at each of the two settings.

### Image analysis

Each still scan image was quantitatively analyzed with the aid of a custom-designed software package (MALUNA™, University of Gottingen, Germany). Distribution of densities within the region of interest was determined according to predefined limits: hyperinflated (HIT) (-900 to -1000 HU), normally aerated (AT) (-500 to -900 HU), poorly aerated (PAT) (-100 to -500 HU) and non-aerated tissue compartments (NAT) (-100 to +100 HU) [14]. The four compartments were expressed as a percentage of the total lung slice weight. The maximal (max), minimal (min), and cyclic variation (Δ, with Δ = max-min) of the four compartments, in each respiratory cycle included within the 8 sec-dCT, were registered and averaged to obtain the corresponding values for the ventilatory setting. R/D was defined as ΔNAT. Based on the appearance of lung morphology on CT, each patient was classified as having focal or non-focal distribution of attenuations [15].

### Statistical analysis

Data were compared by Wilcoxon signed rank test. Analysis was performed with GraphPad Prism version 5.00 for Windows, (GraphPad Software, San Diego, CA, USA). Data are expressed as median (range) and a *P *<0.05 was considered statistically significant.

## Results

We analyzed ten patients (six male), who had a median age of 45 (17 to 87) years old, Acute Physiology and Chronic Health Evaluation II (APACHE II) score of 20 (4 to 29) and sequential organ failure assessment (SOFA) score of 10 (3 to 17) (Table 1). Although all patients had a pulmonary origin of their ARDS, their CT scan showed a bilateral involvement and a non-focal distribution of attenuations. They were on their first 48 hours of mechanical ventilation. At the moment of the study three patients had mild, six had moderate and one had severe ARDS, according to the grading criteria proposed by the recent ARDS consensus in Berlin [16].

**Table 1:** Patient characteristics at baseline.

Patient number	APACHE II	SOFA	PEEP (cmH_2_O)	Vt (ml/kg)	PaO2/FiO2	ARDS onset (days)	Minute ventilation (L/min)	Etiology
1	17	6	16	3.9	148	0	11.1	H1N1
2	5	15	16	6.0	71	0	10.6	Sev Pn
3	23	9	12	9.6	197	0	7.3	Asp
4	23	11	10	6.0	254	0	7.0	H1N1
5	14	9	10	6.1	280	0	9.3	Sev Pn
6	22	17	12	5.6	148	0	8.6	AH
7	29	12	14	7.0	123	0	7.9	Asp
8	15	3	10	6.5	142	0	7.9	H1N1
9	4	3	14	6.0	148	0	7.4	Asp
10	24	15	12	7.6	265	1	8.6	Sev Pn
**min**	4	3	10	3.9	71	0	7.3	
	
**max**	29	17	16	9.6	280	1	11.1	
**median**	20	10	12	6.0	148	0	8.2	

AH, alveolar hemorrhage; APACHE II, Acute Physiology and Chronic Health Evaluation II; ARDS, acute respiratory distress syndrome; Asp, aspiration; H1N1, influenza A H1N1; max, maximal value; min, minimal value; PaO2/FiO2, the ratio of partial pressure of oxygen in arterial blood and fraction of inspired oxygen; PEEP, positive end-expiratory pressure; Sev Pn, severe pneumonia; SOFA, sequential organ failure assessment; Vt, tidal volume.

**Figure 1:**
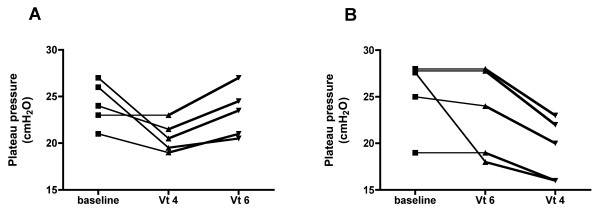
**Individual changes in plateau pressure**. The graphs present individual data of plateau pressure during the protocol, with the five patients randomized to the sequence Vt 4 to Vt 6 (ml/kg) **(A) **and the five patients randomized to the sequence Vt 6 to Vt 4 (ml/kg) **(B)**. Vt, tidal volume.

**Figure 2:**
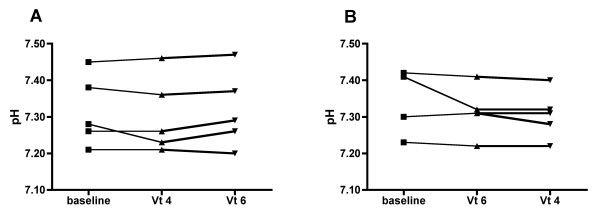
**Individual changes in pH**. The graphs present individual data of pH during the protocol, with the five patients randomized to the sequence Vt 4 to Vt 6 (ml/kg) **(A) **and the five patients randomized to the sequence Vt 6 to Vt 4 (ml/kg) **(B)**. Vt, tidal volume.

When decreasing Vt to 4 ml/kg there was a significant decrease in plateau pressure, mean airway pressure and driving pressure, but no change in respiratory system compliance (Table 2 Figure 1). No patient developed auto-PEEP at any moment of the protocol. There was no change in PaO_2_, or in PaO_2_/FiO_2_. PaCO_2 _and pH increased marginally, but not significantly, when applying the Vt 4 strategy (Table 3, Figure 2). However, no patient developed severe respiratory acidosis or severe hypercapnia during ventilation with Vt 4 ml/kg. The lowest pH observed with Vt 4 ml/kg was 7.22 and the highest PaCO_2 _was 51 mmHg. In addition, there was no significant change in systemic hemodynamics during the study period and all patients completed the whole protocol.

**Table 2:** Respiratory variables.

	Tidal volume		
	**Vt 4**	**Vt 6**	***P***
	
**Tidal volume (ml)**	248 (210-300)	373 (310-450)	0.005
**Respiratory rate (breaths/min)**	37 (28-39)	25 (19-26)	0.005
**Plateau pressure (cmH_2_O)**	21 (16-23)	24 (18-28)	0.008
**Mean airway pressure (cmH_2_O)**	15 (13-20)	16 (14-21)	0.010
**Driving airway pressure (cmH_2_O)**	7 (4-12)	11 (6-18)	0.009
**Compliance _RS _(ml/cmH_2_O)**	39 (18-53)	40 (17-53)	0.234

Values in the table are expressed as median (minimal - maximal). All variables were compared by Wilcoxon signed rank test. Driving airway pressure was calculated as plateau pressure - total positive end-expiratory pressure (PEEP). RS, respiratory system; Vt 4, tidal volume 4 ml/kg predicted body weight (PBW); Vt 6, tidal volume 6 ml/kg PBW.

**Table 3:** Systemic hemodynamics and gas exchange.

	Tidal volume		
	**Vt 4**	**Vt 6 **	***P***
	
**Mean arterial pressure (mmHg)**	79 (65-99)	76 (65-92)	0.725
**Heart rate (beats/min)**	110 (70-135)	106 (68-126)	0.513
**pCO2 (mmHg)**	48 (26-51)	45 (27-51)	0.148
**pH**	7.29 (7.21-7.46)	7.31 (7.20-7.47)	0.105
**pO2 (mmHg)**	86 (54-168)	93 (61-162)	0.358
**EtCO2 (mmHg)**	38 (24-50)	37 (26-45)	0.359
**Alveolar dead space fraction (% Vt)**	13 (2-36)	12 (3-33)	0.820

Values in the table are expressed as median (minimal - maximal). All variables were compared by Wilcoxon signed rank test. EtCO_2, _end tidal CO_2_; pCO_2, _partial pressure of CO_2_; pO_2, _partial pressure of O_2_; Vt 4, tidal volume 4 ml/kg predicted body weight (PBW); Vt 6, tidal volume 6 ml/kg PBW.

**Figure 3:**
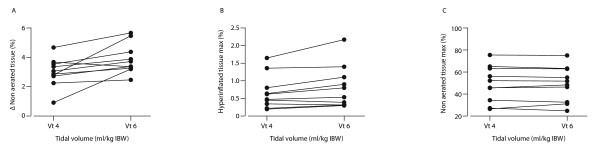
**Individual changes in Δ non-aerated, maximal hyperinflated and maximal non-aerated tissue**. The graphs present Δ non-aerated **(A)**, maximal hyperinflated **(B) **and maximal non-aerated tissue **(C) **(expressed as % of lung tissue weight) for each of the 10 patients with Vt 4 and Vt 6 ml/kg. Vt, tidal volume.

When compared with Vt of 6 ml/kg, Vt 4 significantly decreased ΔNAT and this effect was consistent in all patients (Table 4, Figure 3A). Delta HIT and HITmax also decreased significantly with Vt 4 ml/kg (Table 4 Figure 3B). There was no change in NATmax between both Vts (Figure 3C). We found no relation between ARDS severity and ΔNAT or HIT max.

**Table 4:** Analysis of density fractions of the lungs.

	Tidal volume		
	**Vt 4**	**Vt 6**	***P***
**Maximal non-aereated tissue**	49 (27-76)	50 (26-76)	0.845
**Maximal poorly aerated tissue**	28 (15-44)	28 (15-45)	0.695
**Maximal normally aerated tissue**	24 (10-46)	25 (10-45)	0.275
**Maximal hyperinflated tissue**	0.6 (0.2-1.7)	0.7 (0.3-2.2)	0.013
**Minimal non-aerated tissue**	46 (24-73)	46 (21-73)	0.232
**Minimal poorly aerated tissue**	26 (14-42)	26 (14-39)	0.431
**Minimal normally aerated tissue**	22 (9-45)	22 (9-42)	0.431
**Minimal hyperinflated tissue**	0.2 (0.1-1.1)	0.2 (0.1-1.0)	0.128
**Δ Non-aerated tissue**	2.9 (0.9-4.7)	3.6 (2.5-5.7)	0.006
**Δ Poorly aerated tissue**	2.9 (1.0-4.4)	3.2 (0.4-6.1)	0.322
**Δ Normally aerated tissue**	1.6 (0.0-4.15)	2.1 (0.7-6.7)	0.131
**Δ Hyperinflated tissue**	0.3 (0.1-0.6)	0.5 (0.2-1.2)	0.002

Values in the table correspond to percentage of total lung slice weight and are expressed as median (minimal - maximal). All variables were compared by Wilcoxon signed rank test. Δ, difference between maximal and minimal values. Vt 4, tidal volume 4 ml/kg predicted body weight (PBW); Vt 6, tidal volume 6 ml/kg PBW.

## Discussion

In this preliminary physiologic study we showed in 10 ARDS patients that by decreasing Vt from 6 to 4 ml/kg we were able to reduce cyclic R/D, as well as hyperinflated tissue, plateau pressures and driving pressures. Severe respiratory acidosis and severe hypercapnia could be effectively prevented at Vt 4 ml/kg by increasing respiratory rate and reducing instrumental dead space.

Experimental studies using dynamic CT, subpleural videomicroscopy and PaO_2 _oscillations, have shown evidence that cyclic R/D may occur in ARDS [17-19]. Recently, by applying dynamic CT during mechanical ventilation we provided evidence that cyclic R/D also occurs in ARDS patients [5]. In that study, we demonstrated that decreasing Vt from a high to a low Vt (12 to 6 ml/kg) markedly decreased cyclic R/D. Now, we showed that cyclic R/D may be further decreased by applying a very low Vt, such as 4 ml/kg. Although a 0.7% absolute reduction in R/D may appear as modest, CT scan may systematically underestimate R/D because of the limited resolution of CT images, which defines that the mean CT attenuation measured on each voxel is an average of different alveolar densities. Therefore, voxels showing apparently aerated units at end expiration may contain several collapsed and unstable alveoli mixed with aerated units. In fact, videomicroscopic images obtained in experimental models confirm that unstable alveoli can be adjacent to stable alveoli.

Besides the potential benefit of reducing cyclic R/D, reduction in Vt decreased airway pressures, specifically plateau pressure. Hyperinflated tissue decreased in parallel to plateau pressure. Therefore, these data suggest that a very low Vt may prevent both, overdistension and cyclic R/D. Recently, Terragni *et al*. showed in ARDS patients that decreasing Vt from 6.3 ± 0.2 to 4.2 ± 0.3 ml/kg, was associated to a decrease in tidal hyperinflation, which was paralleled by a decrease in pulmonary inflammation, as assessed by cytokines in bronchoalveolar lavage [6]. However, in their study, Vt reduction was associated to an increase in PEEP levels, and hypercapnia was avoided by the use of extracorporeal CO_2 _removal.

One of the major concerns of using a very low Vt is the risk of hypercapnia. Richecoeur *et al*. showed several years ago that rather simple interventions such as increasing respiratory rate and decreasing instrumental dead space could be effective to control hypercapnia in ARDS patients [20]. Therefore, during ventilation with 4 ml/kg we increased respiratory rate to keep constant minute ventilation. In addition, we reduced instrumental dead space by replacing the HMEF by a heated humidifier. This intervention has been shown to be effective to reduce PaCO_2 _while ventilating ARDS patients with a low Vt strategy [11,21]. Although some authors have suggested that HMEFs should be avoided during ventilation with a Vt of 6 ml/kg [22], this is not a standard practice, and furthermore, several ICUs keep using HMEFs in mechanically ventilated ARDS patients [23,24]. In the present study we kept the HMEF during ventilation with Vt 6, as was the usual practice in our ICU, but we removed it during ventilation with Vt 4. Although this variation in dead space between Vt 6 and Vt 4 limits the possibility of comparing PaCO_2 _and pH with both Vts, the purpose of this part of the study was just to determine if severe hypercapnia and respiratory acidosis could be avoided during ventilation with Vt 4. Although 30 minutes may seem a short period, it has been shown that it is enough to reach a new equilibrium in PaCO_2 _after changes in ventilation [25,26]. Our data suggest that a ventilatory strategy based on a Vt of 4 ml/kg would be feasible in several ARDS patients. However, we acknowledge that only one patient had a severe ARDS, and therefore, future studies should determine whether this intervention can be applied in severe ARDS patients without inducing severe respiratory acidosis or requiring CO_2 _extracorporeal removal.

In the present study we used respiratory rates as high as 39/minute during ventilation with Vt 4. Interestingly, we did not observe auto-PEEP in any patient, probably because expiratory time decreased in the same proportion as Vt and ARDS patients typically have very short time constants. A second concern is that higher respiratory rates may favor VILI, as has been shown in experimental data [27,28]. In fact, although hyperinflation may decrease in magnitude because of the lower Vt, peak inspiratory hyperinflation phenomena occur more frequently because of the compensatory increase in respiratory rate, which could eventually counteract the potential benefit of decreasing Vt. However, the results of the ARDS network tidal volume study suggest that the potential disadvantage of a larger respiratory rate may be largely surpassed by the benefits of a lower Vt [1]. The use of extracorporeal lung support may allow both Vt and respiratory rate to decrease, although it has its own risks.

Another potential disadvantage of further decreasing Vt is a progressive alveolar derecruitment secondary to lower mean and plateau airway pressures. This derecruitment may be prevented by a parallel increase in PEEP levels [6,29,30]. However, as we were interested in the isolated impact of Vt on cyclic R/D, we did not modify PEEP levels. Despite keeping PEEP constant and lowering airway pressures, we did not observe an increase in non-aerated tissue in CT. However, CT images were obtained after only 5 minutes of ventilation. It is possible that after a longer period derecruitment could have been observed [29]. Nevertheless, PaO_2_/FiO_2 _did not change after 30 minutes of ventilation with 4 ml/kg.

### Limitations of the study

As this was designed as a feasibility study, we only tested a short period of ventilation with 4 ml/kg. Before clinical application of such a very low Vt strategy, longer studies should be performed. In addition, although decreasing Vt to 4 ml/kg decreased in 20% cyclic R/D, the absolute magnitude of change was rather small and we did not measure biologic markers of lung injury. A longer protocol would be required to determine if this reduction in cyclic R/D has clinical relevance. Thus, data obtained in this study cannot be directly extrapolated to clinical practice, and although hypothesis-generating, should be interpreted with caution.

A second limitation is the low number of patients studied. We are aware that a larger study may have shown patients who would have developed severe respiratory acidosis or severe hypercapnia with Vt 4 ml/kg. However, our data suggest that cyclic R/D may be decreased with this very low Vt, and could be tolerated in most ARDS patients without inducing severe respiratory acidosis. Even so, as our study population had predominantly moderate ARDS we cannot establish whether patients with more severe forms of ARDS, and who have a larger alveolar dead space, could tolerate being treated with conventional ventilation at Vt 4 ml/kg.

A third limitation is that only pulmonary ARDS patients were included. In our center lung CT scan is not frequently indicated for non-pulmonary ARDS patients so we only had pulmonary ARDS patients included. This limitation determines that our results cannot be generalized to non-pulmonary ARDS patients. However, all patients had a diffuse and bilateral compromise reflecting a real ARDS.

The next challenge is to overcome these limitations by testing a larger number of patients, with more severe forms of ARDS, and for a longer study period. In addition, biologic markers of injury, such as pulmonary and systemic cytokines, must be measured to determine the clinical relevance of the mechanical effects observed.

## Conclusions

In this preliminary physiologic study we observed that decreasing Vt from 6 to 4 ml/kg reduces cyclic R/D, hyperinflation, as well as airway pressures. In addition, we showed that by optimizing instrumental dead space and respiratory rate, it is possible to avoid severe hypercapnia and respiratory acidosis, while ventilating ARDS patients with a Vt of 4 ml/kg PBW. However, these findings must be confirmed in larger studies and complemented with assessment of relevant biomarkers of lung injury, before it can be translated to clinical practice.

## Key messages

• Decreasing tidal volume from 6 to 4 ml/kg reduces cyclic recruitment - derecruitment in ARDS patients

• Decreasing tidal volume from 6 to 4 ml/kg reduces hyperinflation and airway pressures

• The risk of severe hypercapnia and respiratory acidosis after decreasing tidal volume to 4 ml/kg can be minimized by optimizing instrumental dead space and increasing respiratory rate

## Abbreviations

APACHE II: Acute Physiology and Chronic Health Evaluation II; ARDS: acute respiratory distress syndrome; AT: aerated tissue; CT: computed tomography; HIT: hyperinflated tissue; HMEF: heat and moisture exchanging filter; NAT: non-aerated tissue; PAL: poorly aerated tissue; PaO2/FiO2: the ratio of partial pressure of oxygen in arterial blood and fraction of inspired oxygen; PBW: predicted body weight; PEEP: positive end-expiratory pressure; R/D: recruitment - derecruitment; SOFA: sequential organ failure assessment; VILI: ventilator-induced lung injury; Vt: tidal volume.

## Competing interests

The authors declare that they have no competing interests.

## Authors' contributions

JR, GB and AB participated in study design, protocol development and manuscript preparation. JL, MD, MJ and CB participated in data acquisition and analysis, and manuscript revision. All authors have read and approved the manuscript for publication.
